# Assessment of genetic factor and depression interactions for asthma symptom severity in cohorts of childhood and elderly asthmatics

**DOI:** 10.1038/s12276-018-0110-5

**Published:** 2018-07-04

**Authors:** Heung-Woo Park, Woo-Jung Song, Sang-Heon Cho, Michael J. McGeachie, Fernando Martinez, Dave Mauger, Bruce G. Bender, Kelan G. Tantisira

**Affiliations:** 1 Department of Medicine, Channing Division of Network of Medicine, Brigham and Womens Hospital, Harvard Medical School, Boston, MA USA; 2 Department of Internal Medicine, Seoul National University College of Medicine, Seoul, Republic of Korea; 3 Arizona Respiratory Center, University of Arizona, Tucson, AZ USA; 4 Department of Public Health Sciences, Pennsylvania State University College of Medicine, Hershey, PA USA; 5 National Jewish Health, Denver, CO USA; 6 Department of Medicine, Division of Pulmonary and Critical Care Medicine, Brigham and Women’s Hospital, Boston, MA USA

**Keywords:** Genetics, Asthma

## Abstract

It is well known that depression is associated with asthma symptoms. We assessed the combined effects of genetic factors and depression on asthma symptom severity using Bayesian network (BN) analysis. The common 100 top-ranked single-nucleotide polymorphisms (SNPs) were obtained from two genome-wide association studies of symptom severity in two childhood asthmatics trials (CAMP (Childhood Asthma Management Program) and CARE (Childhood Asthma Research and Education)). Using SNPs plus five discretized variables (depression, anxiety, age, sex, and race), we performed BN analysis in 529 CAMP subjects. We identified two nodes (depression and rs4672619 mapping to *ERBB4* (Erb-B2 receptor tyrosine kinase 4)) that were within the Markov neighborhood of the symptom node in the network and then evaluated the interactive effects of depressive status and rs4672619 genotypes on asthma symptom severity. In childhood asthmatics with homozygous reference alleles, severe depression was related to less severe symptoms. However, in childhood asthmatics with heterozygous alleles and homozygous variant alleles, depression and symptom severity showed a positive correlation (interaction permutation *P* value = 0.019). We then tried to evaluate whether the interactive effects that we found were sustained in another independent cohort of elderly asthmatics. Contrary to the findings from childhood asthmatics, elderly asthmatics with homozygous reference alleles showed a positive correlation between depression and symptom severity, and elderly asthmatics with heterozygous alleles and homozygous variant alleles showed a negative correlation (interaction permutation *P* value = 0.003). In conclusion, we have identified a novel SNP, rs4672619, that shows interactive effects with depression on asthma symptom severity in childhood and elderly asthmatics in opposite directions.

## Asthma: How genetic factors and depression affect symptom severity

A novel mutation affects how depression influences the severity of asthma symptoms, with opposite effects in children and the elderly. Depression is known to affect the severity of symptoms experienced by patients with chronic disorders, such as asthma. Heung-Woo Park at Seoul National University College of Medicine in South Korea and coworkers investigated how genetic factors might influence the interaction between depression and asthma. Using previously collected genetic data, the researchers identified a mutation linked with both asthma and depression. In children with the mutation, depression was linked to severe asthma symptoms; children with depression but without the mutation experienced milder symptoms. The researchers observed the opposite relationship in a group of elderly patients with asthma. They conclude that further study of this mutation could illuminate ways to improve asthma management strategies.

## Introduction

A depressive disorder may influence the symptoms present in the course of a chronic illness. For example, the presence of depression was shown to be a stronger predictor of reporting diabetes symptoms than hemoglobin A_1C_ level and was more highly associated with chest pain than objective measures of coronary artery disease severity^1,2^. Asthma is not an exception. Epidemiologic associations between depression and asthma symptoms have been recognized anecdotally for many years^3–5^. Dysregulation in key biologic systems, such as inflammation^6^, neuroendocrine dysregulation^7^, autonomic imbalance causing cholinergic activation^8^, and genetics dysregulation^9^, have been suggested as explanations of this co-occurrence.

A Bayesian network (BN) is a multivariate model of dependency among several variables, and the strength of the relationship between variables is represented by conditional probability distributions associated with each node^10,11^. BNs have been applied in a variety of settings for the purposes of probabilistic prediction, including predictions of asthma exacerbations and control^12,13^ and responses to short-acting bronchodilator and inhaled corticosteroids^14,15^. Given that depression is associated with asthma symptoms, we hypothesized that combining information from multiple SNPs, demographic factors, depression, and anxiety can yield models predictive of symptoms. Using BNs, we developed a predictive model and identified one candidate single-nucleotide polymorphism (SNP), rs4672619, that showed possible combined effects with depression upon asthma symptoms. Rs4672619 is located on the intron of the Erb-B2 receptor tyrosine kinase 4 (*ERBB4*) gene and showed interactive effects with depression on asthma symptom severity in children from the Childhood Asthma Management Program (CAMP) trial^16^. We confirmed that interactions between depression and rs4672619 in predicting asthma symptom severity were also noted in an independent cohort of elderly asthmatics, although the interactions were in the opposite direction.

## Materials and methods

Each study was approved by the Institutional Review Board of the corresponding institution and informed consent was obtained from all study participants. Detailed methods are described in the supplementary material.

### Identifying SNPs associated with asthma symptoms

To obtain reliable SNPs associated with asthma symptoms, we used the results of two genome-wide association study (GWAS) performed in the non-Hispanic white children with asthma enrolled in the CAMP trial (*n* = 438) and in the Childhood Asthma Research and Education (CARE) trials (*n* = 457). At baseline, all participants were asked to rate and score their asthma symptoms during the past 24 h on a diary card. Similar questions were used in both trials, and the symptom scores ranged from 0 (absent) to 3 (severe) in all trials^16–18^. Detailed methods of genome-wide SNP genotyping have been described elsewhere for CAMP^19,20^ and CARE^21^. A common set of SNP genotypes was obtained by imputation in each cohort using MaCH (version 1.0)^22^ and the 1000 Genomes Project EUR reference-phased haplotypes based on Phase 1 low coverage data (20101123 release). SNPs with minor allele frequency <1%, a Hardy–Weinberg equilibrium *P* < 0.001, and/or an imputation quality score <0.3 were excluded, resulting in a set of ~37 million variants per cohort. These data and quality control have been described previously^23^. The association of SNPs with asthma symptom scores at baseline was measured with a linear regression model as implemented in PLINK^24^ using additive genetic models. The regression models were adjusted for age and sex. From results of two GWAS, common SNPs with *P* values <0.05 showing same directionality were obtained. As information on depression and anxiety was available only in the CAMP trial, BN analysis was performed in the CAMP cohort. Among common SNPs, the 100 top-ranked SNPs based on *P* values of CAMP were forwarded to BN analysis.

### BN analysis

We view the BN as primarily a hypothesis-generating step in the analysis. In the CAMP trial, measures of depression and anxiety status were collected at baseline and annually thereafter using the Children’s Depression Inventory^25^ and the Revised Children’s Manifest Anxiety^26^. Higher scores on each of these measures reflect increased problems. For better modeling, we drew 91 subjects with other ethnicities from the CAMP trial in addition to the 438 non-Hispanic white subjects, and resultantly, a total of 529 subjects who had a complete phenotype data set (symptom, depression, and anxiety scores) and genotype data were used for BN analysis. The baseline characteristics are presented in Table 1. We used the open-source software package *bnlearn*^27^ in the statistical and graphical environment R (http://www.r-project.org) for BN analysis. In BN analysis, we included 105 variables: 100 SNPs (homozygous minor alleles and heterozygous and homozygous major alleles); depression score (quartile 1–4); anxiety score (quartile 1–4); age (quartile 1–4); sex (male and female); and race (non-Hispanic white, African American, any Hispanic, and Asian). Prior distributions used were *bnlearn* defaults, and we learned a BN structure using *bnlearn*’s hill-climbing algorithm (a greedy optimizer for the Bayesian posterior probability of the data). Measuring the degree of confidence in a particular graphical feature is a key problem in inferring the network structure^28^. We quantified such a degree of confidence by generating 1000 network structures based on nonparametric bootstrap to the data and by estimating the relative frequency of the feature of interest^28^. Then, we removed undirected arcs and arcs with strength <0.1 from the BN inferred. A previous report showed that removing the arcs with strength <0.1 had a minimal effect on its classification accuracy^29^. According to the Markov blanket property of the BN, only the parents (nodes connected above), children (nodes connected below), and the other parents of those children are required to predict the behavior of a node^30^. We therefore selected the two nodes (depression and rs4672619) that were within the Markov blanket of the symptom node in BN and then performed interaction analysis. To calculate an adjusted *P* value, permutation tests for a linear model as implemented in the “lmPerm” R package^31^ was done.

**Table 1:** Characteristics of childhood and elderly asthmatics

	Childhood asthmatics, *N* = 26	Elderly asthmatics, *N* = 96
Age (years), median (IQR)	8.8 (7.2–10.6)	72.5 (70–77)
Male gender, number (%)	332 (62.7)	38 (39.6)
Symptom score, median (IQR)	0.57 (0.14–1.00)^a^	8 (6–10)^b^
Race, number (%)		
Non-Hispanic white/African American/Hispanic/Asian	389 (73.6)/58 (10.9)/40 (7.6)/42 (7.9)	0 (0)/0 (0)/0 (0)/96 (100)
Cognitive function (score), median (IQR)	NA	27 (24–29)^c^
Anxiety (score), median (IQR)	10 (6–15)	NA
Depression (score), median (IQR)	5 (2–10)	19.5 (16–22)

*IQR* interquartile range, *NA* not applicable

^a^Range 0–3

^b^Range 0–25

^c^Range, 0–30 (any score ≥24 points indicates normal cognition)

### Evaluating the interactive effects in another cohort

We evaluated whether the interactive effects of depression status and rs4672619 genotypes on asthma symptom severity observed in the cohort of childhood asthmatics were sustained in another independent cohort. As no childhood cohort with both depression and genotype data was readily available, our evaluation was done in elderly asthmatics aged 65 years or older from a prospective, observational, and multi-centered cohort in Korea with the purpose of studying the natural history of asthma among elderly people^32^. The baseline characteristics are presented in Table 1. Symptom scores were measured using five questionnaires (range, 0–25; a higher score represents a more severe symptom), and depression status was assessed by the Korean version of the Geriatric Depression Scale Short Form (range, 0–30; a higher score represented more severe depression). Cognitive function was assessed using the Korean version of the Mini-Mental State Examination (range, 0–30; any score ≥27 points indicates a normal cognition). Details for symptom questionnaires are described in the online supplement. rs4672619 was genotyped, and the interactive effects between genotype and depression status on asthma symptom severity were evaluated.

## Results

**Fig. 1:**
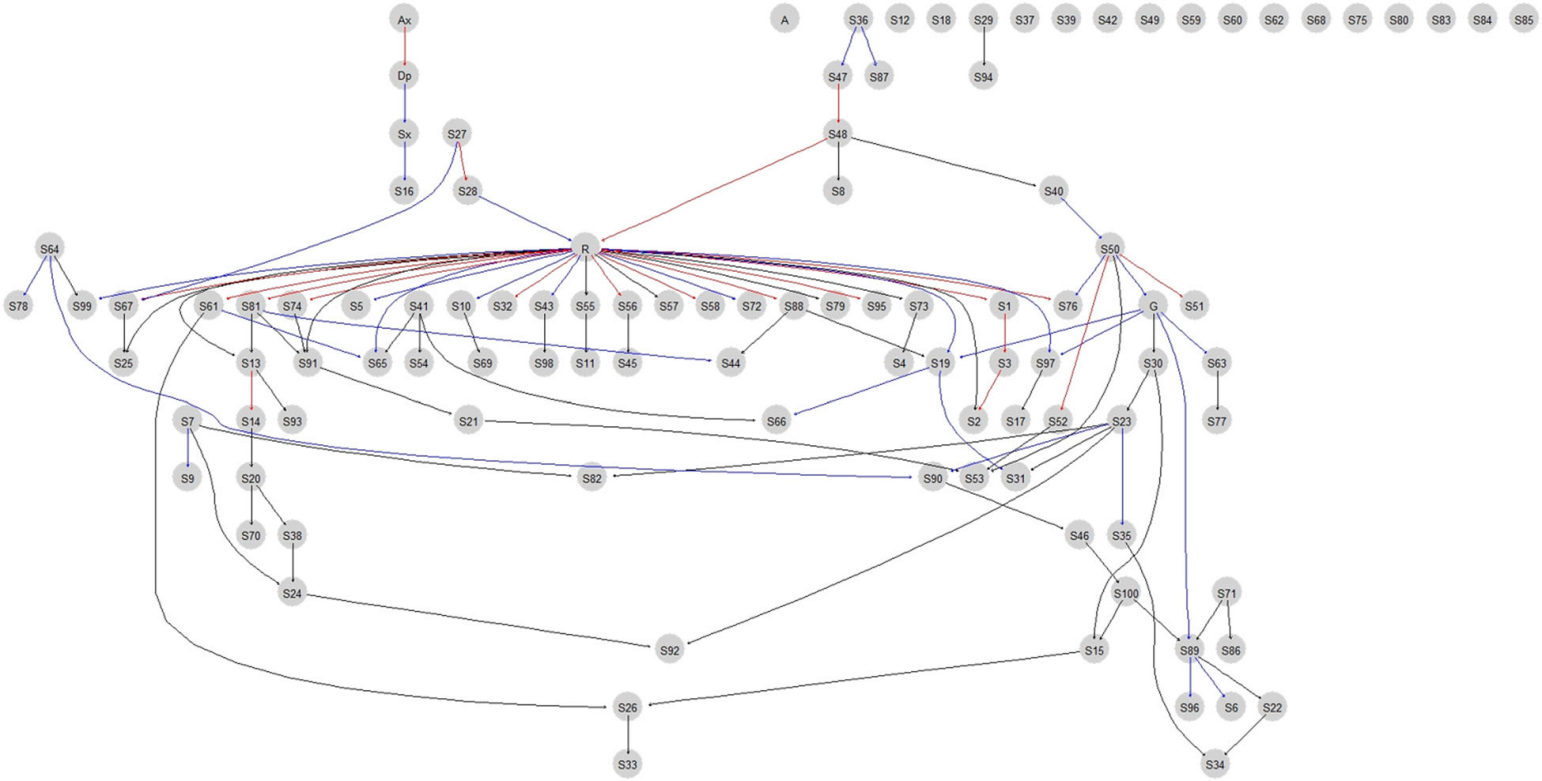
Bayesian network inferred. A Bayesian network learned from the data of childhood asthmatics with a Hill-Climbing algorithm. Edges represent conditional dependencies, and connected nodes represent variables that are conditionally dependent on each other. Red edges represent edges with an arc strength >0.5, blue edges represent edges with an arc strength between 0.2 and 0.5, and black edges represent edges with an arc strength between 0.1 and 0.2. Edges with an arc strength <0.1 were removed from the network. For a certain node, nodes connected above (parent nodes), nodes connected below (children nodes), and the other parent nodes of these children nodes form a Markov blanket, which is required to predict the behavior of that node. Thus, we evaluated the interactive effects of depression severity (parent of the Sx node) and rs4672619 (S16) genotypes (children of the Sx node) on asthma symptom severity (Sx node). A age quartile, Ax anxiety quartile, Dp depression quartile, G gender (male/female), Sx symptom severity quartile, R ethnicity (non-Hispanic white/African American/Hispanic/Asian), and S1-100 top-ranked 100 SNPs (S16, rs4672619)

**Fig. 2:**
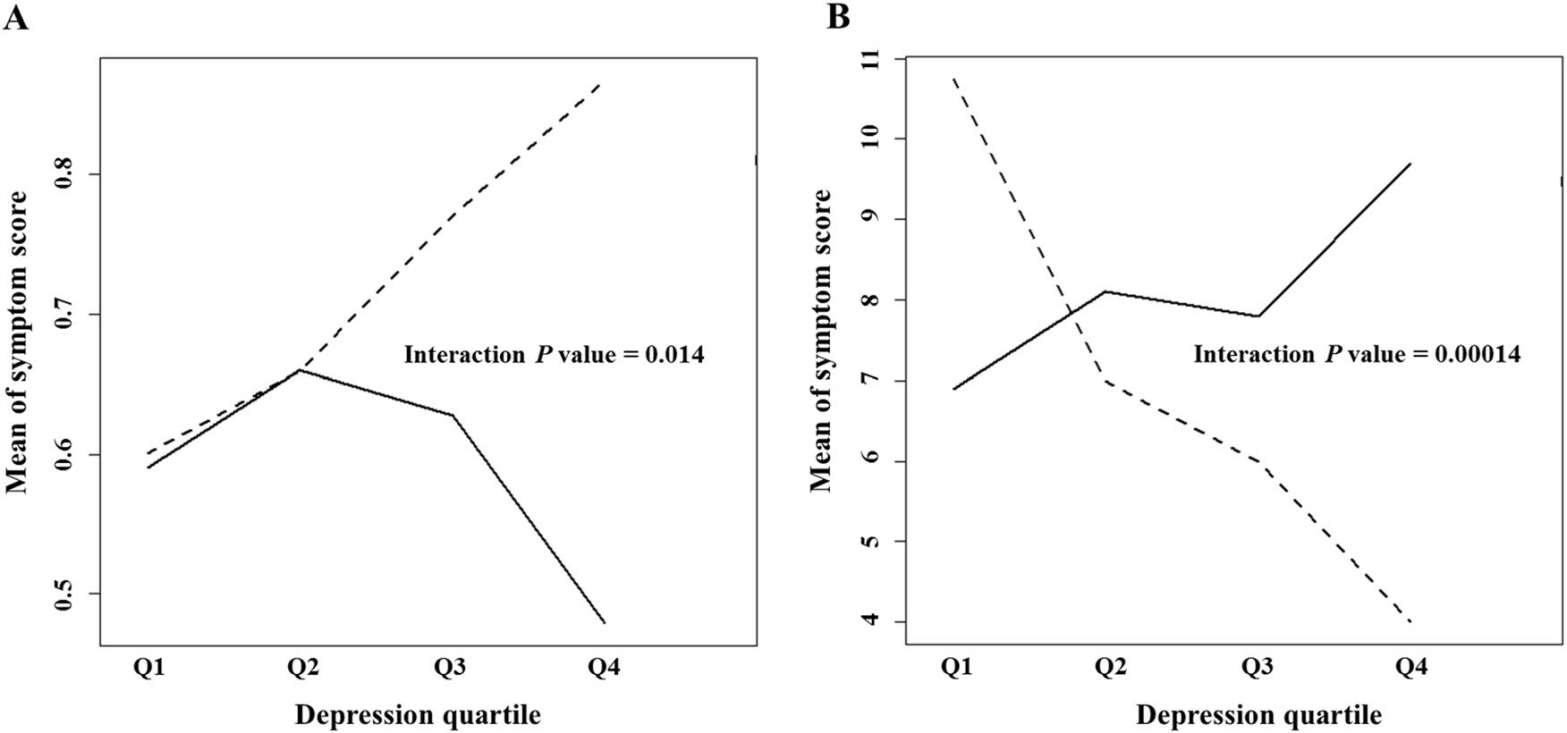
Interactive effects of rs4672619 genotypes and depression severity on asthma symptom severity in cohorts of childhood and elderly asthmatics. **a** Childhood asthmatics. **b** Elderly asthmatics. Solid lines represent subjects with reference homozygous alleles of rs4672619, and dotted lines represent subjects with heterozygous or homozygous variant alleles of rs4672619. Q1 represents mild depression, and Q4 represents severe depression. The severity of asthma symptoms increases as symptom score increases

The lowest *P* value for GWAS analysis was 1.66 × 10^−6^ (rs2141189) in the CAMP trial and 1.52 × 10^−6^ (rs1429146) in the CARE trial. There were 413 common SNPs with *P* values <0.05 with the same directionality. Of these SNPs, the 100 top-ranked SNPs based on the CAMP *P* values (Supplementary Table 1) were selected and forwarded for BN analysis. Figure 1 shows the BN learned from the data of childhood asthmatics. We found two nodes directly attached to the asthma symptom (Sx node in Fig. 1): depression (Dp node in Fig. 1) and rs4672619 (S16 node in Fig. 1). The arc strengths between the Sx and Dp nodes and between the Sx and S16 nodes were 0.329 and 0.207, respectively. We then evaluated the interactive effects of depression severity and rs4672619 genotypes on asthma symptom severity in all CAMP subjects. As shown in Fig. 2a, in childhood asthmatics with homozygous reference alleles (frequency = 86.8%), severe depression was related to less severe asthma symptoms. However, in childhood asthmatics with heterozygous or homozygous variant alleles, depression and asthma symptom severity showed a positive correlation. The *P* values of the main effects were 0.009 for rs4672619 and 0.322 for depression, and the *P* value of the interaction was 0.014 (permutation *P* value = 0.019). rs4672619 was genotyped in 96 Korean elderly asthmatics, and we also found that the directions of correlations between depression status and asthma symptom severity differed according to rs4672619 genotypes (Fig. 2b). However, the direction of correlation was opposite to that of childhood asthmatics (Fig. 2). That is, elderly asthmatics with homozygous reference alleles (frequency = 92.4%) showed a positive correlation between depression and symptom severity. The *P* values of the main effects were 0.177 for rs4672619 and 0.125 for depression, and the *P* value of the interaction was 0.00014 (permutation *P* value = 0.003).

## Discussion

Precise symptom perception would be an important component of asthma management, allowing asthmatics to use rescue medicine or seek medical help in a timely manner^33^. However, there may be a discrepancy between asthma symptom perception and lung function^34^. Therefore, asthma symptoms are a unique domain that should be evaluated for the proper management of asthma^35^. β2 Adrenergic receptor gene methylation was reported to be associated with decreased asthma severity in inner-city schoolchildren^36^, and genetic components are known to be involved in asthma symptom responses to medications^37,38^. In addition, a recent report showed that psychosocial factors, such as harsh parent–child conflict^39^ or targeted rejection^40^, caused severe symptoms in children with asthma by decreasing anti-inflammatory gene expression. These findings motivated us to assess the interactive effects of multiple SNPs mapped to multiple genes and depression severity on asthma symptoms. Using BNs to guide our investigation, we found that rs4672619 was directly attached to the symptom node. Further analysis has shown that rs4672619 genotypes showed significant interactive effects with depression severity on symptom severity in both childhood and elderly asthmatics.

rs4672619 is on an intron of the *ERBB4* gene. *ERBB4*, along with its ligand neuregulin-1, is known to contribute to the pathophysiology of schizophrenia and bipolar disorders^41,42^. A recent Bayesian modeling approach identified several variants in *ERBB4* with strong evidence for associations with childhood asthma^43^. Recent increases in the understanding of dyspnea, one of the important asthma symptoms, suggest that there may be interactions between biopsychological factors and dyspnea perception^44^. *ERBB4* regulates a thalamic reticular nucleus to cortical inputs at levels that can support sensory selection while allowing behavioral flexibility^45^. In addition, crosstalk between the ErbB network and steroid hormone signaling pathway has been well known^46^ and severe, steroid-dependent asthmatics had depression more often than non-steroid-dependent asthmatics^47^. Taken together, rs4672619 is possibly involved in interactions between depression and asthma symptom severity by modulating symptom perception and steroid action.

One of the interesting findings of this study was that the direction of interactive effects of rs4672619 genotypes with depression severity on asthma symptoms was opposite between our childhood and elderly cohorts. The childhood asthmatics carrying two reference alleles showed significantly negative correlations between depression and symptoms scores, whereas elderly asthmatics with these alleles showed significantly positive correlations. Cognitive dysfunction, which is frequently encountered in elderly asthmatics^48^, might affect asthma symptom presentation. However, we found that the Mini-Mental State Examination scores of all elderly asthmatics were greater than 24 (Table 1), which suggested that they were cognitively competent^49^. Many investigators have agreed that elderly asthma differs considerably from non-elderly asthma and that aging lungs^50^, physiologic changes^51^, and immunosenescence^52^ are possible contributing factors. Likewise, the different interactive effects of rs4672619 genotypes and depression severity on asthma symptom severity between childhood and elderly asthmatics might come from the factors mentioned above. It is also possible that asthma symptom perception may be influenced by age, and a specific genetic mechanism regulating childhood asthma symptom severity may not carry over to old age. To date, no study has evaluated differences in the perception of asthma symptoms in childhood and elderly asthmatics. However, for childhood asthmatics, it was reported that adolescents (13–18 years) were more accurate in perceiving symptoms than school-age children (6–12 years)^31^ and that the accuracy for perceiving symptoms increased with age in children aged 7 to 17 years^53^. In our previous study, we found that genetic mechanisms underlying the symptomatic response to inhaled corticosteroids might be different between childhood and adult asthmatics^36^. Interestingly, recent reports showed that some genetic variations in *ERBB4* showed age-by-genotype interactions on cortical brain morphology in a cohort of 3–20 year olds^54^, and age-dependent differences in *ERBB4* expression were found in the brains of autistic patients^55^. These findings suggest that the genetic effects of *ERBB4* may be different between childhood and elderly asthmatics, although we do not know whether the same differences exist in the lung.

Another plausible explanation is that the different interactive effects are due to ethnicity differences. Childhood asthmatics in this study were predominantly non-Hispanic white patients, and whereas elderly asthmatics were only Asian patients. Allele reversal at shared risk loci, the so-called flip-flop phenomenon, can be attributed to differences in the underlying genomic architectures at these loci according to ethnicity differences^56^. For example, a previous report showed that variants of *ENND1B* were associated with asthma in children, but the association in the African Americans was linked to the opposite allele of that associated with asthma in subjects of European ancestry^57^. Very little is known about ethnicity–gene interactions for *ERBB4*. Only one small-scale study conducted in non-Hispanic white and African-American schizophrenia patients reported that disease-associated SNPs on *ERBB4* showed no ethnicity–genotype effects on *ERBB4* splice-variant expression levels in the brain ^58^.

There are a few potential limitations in generalizing our findings. Inevitably, the main effect and interaction effect are often correlated. It is possible that the significant effect of rs4672619 genotypes might have affected the interactive effect of depression severity and rs4672619 genotypes on asthma symptom severity in childhood asthmatics. Next, we could not replicate our results in another pertinent cohort of childhood asthmatics. The opposite directions of the correlations in childhood and elderly asthmatics was interesting. However, we cannot completely exclude the possibility that our results may be due to chance alone (false-positive associations). In addition, further mechanistic studies are needed to confirm the age-specific or ethnicity-specific effects of rs4672619 on the *ERBB4* gene. An incorporation of prior biological knowledge would help us overcome the reconstruction accuracy due to the complex nature of the network and the noise inherent in the data.

In conclusion, we have identified rs4672619, which showed significant interactive effects with depression severity on asthma symptom severity in both childhood and elderly asthmatics in opposite directions. This study is the first to show significant interactions between genetic factors and depression severity for asthma symptom severity. Our findings suggest that different strategies to decrease asthma symptom severity may be needed in these distinct age subsets of asthmatics.

## Electronic supplementary material

Supplementary material
