# Supplementary material for: Assessment of genetic factor and depression interactions for asthma symptom severity in cohorts of childhood and elderly asthmatics
Source: Exp Mol Med. 2018 Jul 4;50(7):1–7. doi: 10.1038/s12276-018-0110-5 (PMC6031659; doi:10.1038/s12276-018-0110-5)
Supplement: Supplementary file 1 — Supplementary material [file 12276_2018_110_MOESM1_ESM.docx]

**Assessment of genetic factor and depression interactions for asthma symptom severity in cohorts of childhood and elderly asthmatics**

**Supplementary material**

**Methods**

***Characteristics of the clinical trials of childhood asthmatics and symptom questionnaires***

Childhood Asthma Management Program (CAMP) : This population is composed of white subjects from a clinical trial that followed 1,041 asthmatic children for 4-6 years (4.3 years on average) (1). Stringent inclusion criteria ensured that participants had mild to moderate asthma, which was assessed as having asthma symptoms at least twice per week, using asthma medication daily, or using an inhaled bronchodilator twice per week for six or more months of the year prior to recruitment.^1^ Primary clinical trial outcomes have been published (2). As part of the clinical trial, individuals were randomly assigned to one of three treatment arms, budesonide (an inhaled corticosteroid), nedocromil, or placebo.

Childhood Asthma Research and Education (CARE): Subjects from the following CARE trials were used for the current study: Characterizing the Response to a Leukotriene Receptor Antagonist and an Inhaled Corticosteroid (CLIC) (3) and Pediatric Asthma Controller Trial (PACT) (4) In CLIC, children (ages 6-17 years) with mild-to-moderate persistent asthma were randomized to one of 2 crossover sequences, including 8 weeks of an ICS, fluticasone propionate (100 µg twice daily), and 8 weeks of montelukast (5-10 mg nightly depending on age). In PACT, children (ages 6-14 years) with mild-to-moderate persistent asthma on the basis of symptoms, and with FEV1 ≥ 80% predicted and methacholine FEV1 PC20 ≤ 12.5mg/mL, were randomized to 1 of 3 double-blind 48-week treatments: fluticasone 100 µg twice daily (fluticasone monotherapy), fluticasone 100 µg/salmeterol 50 mg in the morning and salmeterol 50 mg in the evening (PACT combination), and montelukast 5 mg in the evening.

Symptom questionnaires: For the younger enrollees, CAMP expects and encourages parents to supervise administration of study medication and completion of diary cards (2). As the children approach and enter adolescence, the coordinators and educators at the clinic make it a priority to teach them to keep the diaries themselves and to take increasing responsibility for their medication. Parental involvement is still needed, but clinics start emphasizing partnerships to create environments in which the children can carry out the responsibility. In CARE, the parents were asked to keep a daily diary of their child’s asthma signs and symptoms, use of asthma and study medications, and contact with physicians for asthma (5). Guidance to answer in the CAMP trials were as follows: *Enter the code that best describe your asthma during the past 24 hours. An asthma episode is a single period of 1 or more asthma "stop signs", such as wheezing, coughing, chest tightness, or shortness of breath. 0 = no asthma episodes; 1 = 1~3 asthma episode, each lasting 2 hours or less (all mild); 2 = 4 or more mild asthma episode, or 1 or more asthma episodes that temporarily interfered with activity, play, school, or sleep; 3 = 1 or more asthma episodes lasting longer than 2 hours, or resulting in shortening normal activity, or seeing a doctor, or going to a hospital*. Guidance to answer in the CARE was as follows: *Enter the code that best describe your asthma during the past 24 hours. Symptom severity rating scale. 0 = Absent (no symptom); 1 = Mild (symptom was minimally troublesome, i.e. not sufficient to interfere with normal daily activity or sleep); 2 = Moderate (symptom was sufficiently troublesome to interfere with normal daily activity or sleep); 3 = Severe (symptom was so severe as to prevent normal activity and/or sleep).*

***Symptom questionnaires in the cohort of elderly asthmatics***

An asthma symptom score was evaluated using self-reported questionnaires composed of five questions; “*During the past 2 weeks, how often have you had chest tightness*?”, “*During the past 2 weeks, how often have you had shortness of breath*?”, “*During the past 4 weeks, how often have you had wheezy breathing sound*?”, “*During the past 2 weeks, how often did your asthma symptoms (wheezing, coughing, shortness of breath, chest tightness or pain) wake you up at night or earlier than usual in the morning*?”, and “*In the past 2 weeks, how much of the time did your asthma keep you from getting as much done at work, school or at home*?” In response to each question, elderly asthmatics were asked to rate their symptoms from 1 to 5 (1, *None of the time*; 2, *A little of the time*; 3, *Some of the time*; 4, *Most of the time*; 5, *All of the time*). Total score ranged from 0 to 25 and a higher score represented a more severe symptom.

**Supplementary references**

E1. Childhood Asthma Management Program Research Group. The Childhood Asthma Management Program (CAMP): design, rationale, and methods. *Control Clin Trials* 1999;20:91-120.

E2. The Childhood Asthma Management Program (CAMP): design, rationale, and methods. Childhood Asthma Management Program Research Group. *Control Clin Trials* 1999;20:91-120.

E3. Szefler SJ, Phillips BR, Martinez FD, Chinchilli VM, Lemanske RF, Strunk RC, Zeiger RS, Larsen G, Spahn JD, Bacharier LB, Bloomberg GR, Guilbert TW, Heldt G, Morgan WJ, Moss MH, Sorkness CA, Taussig LM. Characterization of within-subject responses to fluticasone and montelukast in childhood asthma. *J Allergy Clin Immunol* 2005;115:233-242.

E4. Sorkness CA, Lemanske RF Jr, Mauger DT, Boehmer SJ, Chinchilli VM, Martinez FD, Strunk RC, Szefler SJ, Zeiger RS, Bacharier LB, Bloomberg GR, Covar RA, Guilbert TW, Heldt G, Larsen G, Mellon MH, Morgan WJ, Moss MH, Spahn JD, Taussig LM; Childhood Asthma Research and Education Network of the National Heart, Lung, and Blood Institute. Long-term comparison of 3 controller regimens for mildmoderate persistent childhood asthma: the Pediatric Asthma Controller trial. *J Allergy Clin Immunol* 2007;119:64-72.

E5. Guilbert TW, Morgan WJ, Krawiec M, Lemanske RF Jr, Sorkness C, Szefler SJ, Larsen G, Spahn JD, Zeiger RS, Heldt G, Strunk RC, Bacharier LB, Bloomberg GR, Chinchilli VM, Boehmer SJ, Mauger EA, Mauger DT, Taussig LM, Martinez FD; Prevention of Early Asthma in Kids Study,Childhood. The prevention of Early Asthma in Kids Study, Childhood Asthma Research and Education Network. The Prevention of Early Asthma in Kids study: design, rationale and methods for the Childhood Asthma Research and Education network. *Control Clin Trials* 2004;25:286-310.

E6. Tantisira KG, Lasky-Su J, Harada M, Murphy A, Litonjua AA, Himes BE, Lange C, Lazarus R, Sylvia J, Klanderman B, Duan QL, Qiu W, Hirota T, Martinez FD, Mauger D, Sorkness C, Szefler S, Lazarus SC, Lemanske RF Jr, Peters SP, Lima JJ, Nakamura Y, Tamari M, Weiss ST. Genomewide association between *GLCCI1* and response to glucocorticoid therapy in asthma. *N Engl J Med* 2011;365:1173-1183.

**Supplementary Table 1. The top 100-ranked SNPs identified in GWAS of the cohort of childhood asthmatics**

|  | SNP | Chromosome | Base Pair | CAMP | | CARE | |
| --- | --- | --- | --- | --- | --- | --- | --- |
|  |  |  |  | Beta | P | Beta | P |
| S45 | rs9389694 | 6 | 139909500 | -0.1925 | 0.0001669 | -0.09719 | 0.03056 |
| S7 | rs883853 | 2 | 9872049 | 0.1214 | 0.0003372 | 0.05956 | 0.03801 |
| S52 | rs9692729 | 8 | 20930734 | 0.1209 | 0.0004064 | 0.06107 | 0.03352 |
| S88 | rs725543 | 17 | 66848285 | 0.1149 | 0.001387 | 0.0664 | 0.022 |
| **S16** | **rs4672619** | **2** | **212165440** | **0.1418** | **0.00284** | **0.09222** | **0.03723** |
| S24 | rs9850039 | 3 | 21689499 | -0.1266 | 0.003196 | -0.088 | 0.01239 |
| S40 | rs17111695 | 5 | 150412639 | 0.1291 | 0.003261 | 0.08425 | 0.01796 |
| S37 | rs1514867 | 5 | 17797421 | -0.1375 | 0.003916 | -0.08897 | 0.02507 |
| S50 | rs1402651 | 8 | 20921503 | 0.09945 | 0.003969 | 0.05904 | 0.03957 |
| S87 | rs4785839 | 16 | 64168259 | 0.1043 | 0.003984 | 0.07281 | 0.0204 |
| S29 | rs4681124 | 3 | 147906030 | -0.1851 | 0.004072 | -0.1741 | 0.00664 |
| S31 | rs922521 | 4 | 8258710 | 0.0963 | 0.00443 | 0.05585 | 0.04809 |
| S35 | rs2048075 | 4 | 178491898 | -0.1004 | 0.004507 | -0.06014 | 0.03228 |
| S46 | rs10264676 | 7 | 29431390 | 0.1232 | 0.004728 | 0.06775 | 0.04689 |
| S90 | rs4800180 | 18 | 20040486 | 0.1204 | 0.005034 | 0.07713 | 0.03152 |
| S12 | rs16844322 | 2 | 141078464 | -0.1322 | 0.005932 | -0.09789 | 0.01423 |
| S19 | rs7597072 | 2 | 230068331 | 0.0896 | 0.006378 | 0.05541 | 0.04167 |
| S27 | rs6764100 | 3 | 146947240 | -0.1298 | 0.006704 | -0.1076 | 0.008037 |
| S99 | rs9980272 | 21 | 42002476 | 0.1344 | 0.006904 | 0.08044 | 0.03089 |
| S55 | rs7836768 | 8 | 65550021 | 0.09385 | 0.008028 | 0.06603 | 0.0272 |
| S49 | rs2976955 | 8 | 8280470 | 0.1632 | 0.009048 | 0.0944 | 0.04993 |
| S91 | rs4800613 | 18 | 20861721 | -0.0974 | 0.009299 | -0.06295 | 0.04842 |
| S98 | rs2284550 | 21 | 33540155 | -0.08966 | 0.009661 | -0.0809 | 0.00419 |
| S66 | rs1871445 | 10 | 62218680 | -0.0909 | 0.009821 | -0.0645 | 0.03558 |
| S70 | rs10894710 | 11 | 132980228 | 0.1611 | 0.01027 | 0.1158 | 0.04149 |
| S83 | rs573075 | 15 | 23976155 | -0.08804 | 0.01037 | -0.07626 | 0.007344 |
| S47 | rs1468157 | 7 | 126143387 | 0.1122 | 0.01044 | 0.09976 | 0.009632 |
| S6 | rs11891922 | 2 | 9567417 | 0.08591 | 0.01056 | 0.05929 | 0.04262 |
| S58 | rs524888 | 9 | 6449274 | 0.08628 | 0.01057 | 0.06378 | 0.02043 |
| S26 | rs340063 | 3 | 107198700 | -0.09958 | 0.01077 | -0.0615 | 0.04908 |
| S33 | rs9998533 | 4 | 161316842 | 0.1061 | 0.01105 | 0.08414 | 0.01318 |
| S95 | rs10500304 | 19 | 56276410 | 0.08834 | 0.01145 | 0.0578 | 0.04608 |
| S81 | rs175718 | 14 | 75056332 | 0.08191 | 0.01205 | 0.06567 | 0.01836 |
| S97 | rs2830593 | 21 | 27268050 | 0.09815 | 0.01247 | 0.1104 | 0.001244 |
| S30 | rs7610374 | 3 | 156864828 | 0.1106 | 0.01255 | 0.08071 | 0.02941 |
| S73 | rs2306851 | 12 | 53534473 | -0.09676 | 0.0131 | -0.07999 | 0.01496 |
| S68 | rs4572098 | 11 | 95560647 | 0.1324 | 0.01329 | 0.07857 | 0.04503 |
| S4 | rs11240594 | 1 | 204162858 | 0.1116 | 0.01343 | 0.1154 | 0.001735 |
| S41 | rs190935 | 5 | 168517041 | -0.1028 | 0.01363 | -0.1002 | 0.009642 |
| S94 | rs2081893 | 19 | 52739090 | 0.1339 | 0.01365 | 0.09826 | 0.03618 |
| S64 | rs10508468 | 10 | 13958759 | -0.08555 | 0.01391 | -0.07301 | 0.01151 |
| S48 | rs10256873 | 7 | 126155528 | 0.0843 | 0.01426 | 0.06826 | 0.01978 |
| S67 | rs1232186 | 11 | 31579110 | -0.0822 | 0.01466 | -0.05738 | 0.04592 |
| S92 | rs17695084 | 18 | 54806930 | 0.08672 | 0.01474 | 0.08086 | 0.007126 |
| S71 | rs4242889 | 12 | 8507076 | 0.1019 | 0.01496 | 0.07206 | 0.04278 |
| S65 | rs7898560 | 10 | 36647886 | -0.1041 | 0.01508 | -0.07703 | 0.02332 |
| R80 | rs1548687 | 14 | 72028222 | 0.0918 | 0.0151 | 0.0665 | 0.03118 |
| R56 | rs6651329 | 8 | 78368140 | -0.08011 | 0.01584 | -0.06715 | 0.0188 |
| S14 | rs7605998 | 2 | 207511793 | 0.0819 | 0.01639 | 0.06104 | 0.04005 |
| S51 | rs7821595 | 8 | 20925819 | -0.08311 | 0.01711 | -0.0804 | 0.004192 |
| S86 | rs8034011 | 15 | 92997089 | 0.1233 | 0.0173 | 0.1164 | 0.01246 |
| S44 | rs7759361 | 6 | 103904680 | -0.08226 | 0.01758 | -0.0596 | 0.03353 |
| S54 | rs16880153 | 8 | 32881490 | 0.1262 | 0.01864 | 0.1261 | 0.004751 |
| S1 | rs2480775 | 1 | 10529583 | 0.097 | 0.01989 | 0.09572 | 0.008433 |
| S74 | rs838923 | 12 | 123798850 | 0.08074 | 0.02 | 0.0679 | 0.0149 |
| S9 | rs7593229 | 2 | 55534000 | -0.08396 | 0.02011 | -0.08284 | 0.009902 |
| S17 | rs1438831 | 2 | 224614559 | 0.08345 | 0.02035 | 0.07144 | 0.02438 |
| S28 | rs2687876 | 3 | 146973836 | -0.07898 | 0.02074 | -0.09225 | 0.002618 |
| S3 | rs10864463 | 1 | 10536147 | 0.09621 | 0.02083 | 0.09133 | 0.0121 |
| S23 | rs7629800 | 3 | 21629922 | 0.07851 | 0.02149 | 0.05812 | 0.04137 |
| S63 | rs2780238 | 9 | 119793334 | 0.07803 | 0.02158 | 0.0762 | 0.01369 |
| S42 | rs9295967 | 6 | 31291999 | -0.09242 | 0.02162 | -0.07467 | 0.03611 |
| S20 | rs3792106 | 2 | 233855479 | -0.07898 | 0.02164 | -0.06355 | 0.0245 |
| S78 | rs17181636 | 14 | 41266725 | -0.0899 | 0.02166 | -0.06924 | 0.03768 |
| S69 | rs11218802 | 11 | 122146466 | 0.08987 | 0.02173 | 0.07546 | 0.02899 |
| S10 | rs842764 | 2 | 60726355 | 0.07563 | 0.0219 | 0.06077 | 0.03928 |
| S84 | rs10519147 | 15 | 45895411 | -0.111 | 0.02273 | -0.08157 | 0.0485 |
| S22 | rs9864595 | 3 | 4395255 | 0.0925 | 0.02279 | 0.06713 | 0.04014 |
| S8 | rs11885902 | 2 | 14979318 | 0.08968 | 0.02287 | 0.08912 | 0.005501 |
| S43 | rs2499450 | 6 | 39736310 | 0.1289 | 0.02344 | 0.09676 | 0.03125 |
| S96 | rs6096781 | 20 | 35960629 | -0.1465 | 0.0237 | -0.1393 | 0.04015 |
| S2 | rs944191 | 1 | 10530038 | 0.0942 | 0.02387 | 0.09593 | 0.008221 |
| S93 | rs12605879 | 18 | 65516906 | -0.07758 | 0.02427 | -0.05814 | 0.04041 |
| S25 | rs734184 | 3 | 59529936 | 0.07807 | 0.02456 | 0.06215 | 0.02515 |
| S21 | rs344404 | 3 | 2967622 | 0.0768 | 0.025 | 0.0766 | 0.00598 |
| S79 | rs11628040 | 14 | 55328750 | -0.08844 | 0.025 | -0.07685 | 0.00796 |
| S72 | rs4761907 | 12 | 51166364 | -0.08952 | 0.0253 | -0.0637 | 0.04891 |
| S60 | rs1907438 | 9 | 108388801 | 0.1655 | 0.02572 | 0.1456 | 0.007557 |
| S53 | rs898647 | 8 | 21064102 | 0.07344 | 0.02586 | 0.06134 | 0.03258 |
| S59 | rs13285335 | 9 | 72607495 | -0.109 | 0.02596 | -0.09076 | 0.03874 |
| S15 | rs6712328 | 2 | 209431703 | -0.07793 | 0.02655 | -0.09719 | 0.002599 |
| S82 | rs12589656 | 14 | 79262615 | 0.1016 | 0.02674 | 0.09733 | 0.007613 |
| S18 | rs4973216 | 2 | 228269497 | 0.07484 | 0.02682 | 0.07068 | 0.0154 |
| S76 | rs4941980 | 13 | 39956857 | -0.1012 | 0.02735 | -0.1034 | 0.006762 |
| S13 | rs11897520 | 2 | 207456367 | 0.07733 | 0.02756 | 0.06358 | 0.02665 |
| S100 | rs132470 | 22 | 43548040 | 0.1078 | 0.02851 | 0.08017 | 0.04062 |
| S5 | rs2042525 | 1 | 215805659 | 0.08108 | 0.02854 | 0.09279 | 0.006171 |
| S85 | rs10518995 | 15 | 57083368 | 0.1612 | 0.02904 | 0.1944 | 0.002103 |
| S89 | rs16954178 | 18 | 8784092 | 0.1749 | 0.02961 | 0.1322 | 0.02659 |
| S36 | rs16901347 | 5 | 11225773 | 0.1 | 0.02967 | 0.07806 | 0.04078 |
| S38 | rs6872530 | 5 | 80348873 | -0.09061 | 0.03004 | -0.1204 | 0.003145 |
| S32 | rs2601573 | 4 | 18563753 | 0.175 | 0.03028 | 0.1534 | 0.03794 |
| S11 | rs4662981 | 2 | 130176585 | -0.07438 | 0.0305 | -0.06466 | 0.02308 |
| S61 | rs4978760 | 9 | 110700971 | 0.0707 | 0.0306 | 0.06062 | 0.03435 |
| S57 | rs12548312 | 8 | 79967700 | -0.09801 | 0.03087 | -0.1095 | 0.001316 |
| S39 | rs10515451 | 5 | 112322529 | 0.1253 | 0.03114 | 0.09646 | 0.03917 |
| S75 | rs1170932 | 13 | 35711522 | 0.09282 | 0.03166 | 0.1221 | 0.001346 |
| S62 | rs10980170 | 9 | 111862004 | 0.1204 | 0.03197 | 0.1615 | 9.32E-05 |
| S77 | rs285669 | 13 | 76023588 | 0.07516 | 0.03293 | 0.06922 | 0.01934 |
| S34 | rs17060468 | 4 | 175486008 | 0.08845 | 0.03299 | 0.07884 | 0.02073 |
